# An evaluation of SOX2 and hTERC gene amplifications as screening markers in oral and oropharyngeal squamous cell carcinomas

**DOI:** 10.1186/1755-8166-7-5

**Published:** 2014-01-10

**Authors:** Nadja Kokalj Vokač, Bogdan Čizmarević, Andreja Zagorac, Boris Zagradišnik, Boštjan Lanišnik

**Affiliations:** 1 Laboratory of Medical Genetics, University Medical Centre Maribor, Maribor, Slovenia; 2 Medical Faculty, University of Maribor, Maribor, Slovenia; 3 Departement of Otorinolaringology, University Medical Centre Maribor, Maribor, Slovenia

**Keywords:** SOX and hTERC gene amplifications, Brush biopsy, Oral, Oropharyngeal squamous cell carcinoma

## Abstract

**Background:**

Oral and oropharyngeal squamous cell carcinomas (OSCC) are among the most common cancers. The poor survival rate among oral cancer patients can be attributed to several factors, one of them being lack of early detection. A key approach to this problem would be to detect potentially malignant lesion at their early stage. Using the FISH technique, oral brush cytology slides can be an easy and rapid screening approach for malignant cell detection. The present study was designed to detect hTERC and SOX2 amplifications in OSSC exfoliative tumor cells and evaluate whether those two gene amplifications might serve as a supportive biomarker in early detection and diagnosis of oral and oropharyngeal SCC.

**Results:**

Brush biopsies were collected from exophytic and exulcerated oral and oropharyngeal lesions of the oral cavity of 71 patients and 22 healthy controls. FISH techniques using a TERC-specific DNA probe and a SOX2 DNA specific probe both combined with a centromere 3-specific control probe was performed on the cytology slides. A 100 squamous epithelial cell nuclei of the smears per slide were analysed. As abnormal FISH pattern were considered amplified and polyploid patterns.

From 71 brush biopsies of oropharynx and other locations in oral cavity analysed by FISH 49 were considered to be abnormal (69%). The over representation of polyploidy and/or TERC/SOX2 amplification in tumour samples was statistically significant when compared to controls (p = 0.01).

**Conclusion:**

SOX2 and TERC gene amplifications are common in all squamous cell carcinomas and their detection in early stages could be crucial for early detection and more accurate prognosis. Our study strongly suggests that early detection by FISH on cytobrushed samples could be a possible non-invasive screening method even before a tissue biopsy is performed.

## Background

Oral and oropharyngeal squamous cell carcinomas (OSCCs) are among the most common cancers, and a major cause of moratlity, with approximately 400,000 new cases and 200,000 deaths reported worldwide every year. For more than a half of OSSC patients the overall survival rate is only five years [1]. The poor survival rate among oral cancer patients can be attributed to several factors, including a lack of early detection. The established criteria of disease evaluation are biopsy diagnosis, lymph node involvement and stage, although they do not sufficiently account for the variability in clinical outcome. A key solution to this problem would be to detect potentially malignant lesions at an early stage. Tissue biopsy is performed only when the lesions become symptomatic. New methods are needed for the earlier detection of malignant cells, as are genetic markers for assessing disease aggressiveness and prognosis. Exfoliative cytology is an easy, non-invasive procedure and hence could be performed even on the suspicious lesion. Performing FISH on oral brush cytology slides can be a simple and rapid screening tool for malignant cell detection. In our study, the rate of cancer cell detection was evaluated on OSCC cancer samples using this noninvasive technique.

During the last two decades, several genome screening approaches have been developed, including chromosome comparative genomic hybridization (CGH) and array-CGH. These techniques have made it possible to classify chromosomal aberations at a high resolution, which can reveal frequent copy number gains and losses. Many such copy number aberrations harbor oncogenes or tumor suppressor genes and have therefore emerged as predictive and prognostic markers for tumors. Gains of chromosome arms 1q, 3q, 3q 5p, 7p, 7q, 8q, 9q, 11q, 14q, 16p, 19q, and 20q and losses in 2p, 3p, 3p, 4q, 8p, 10p, 16q and 18q have been detected in OSCC samples, and genes located in those regions have been suggested as putative cancer-associated genes that might be altered during oral carcinogenesis [2-5]. Various proto-oncogene amplifications within critical regions have been detected, including TERT at 5p15, EGFR at 7p12, MYC at 8q24, and CCND1 at 11q13 [2,6]. A gain in the 3q26 region, which contains the hTERC and SOX2 genes, is frequently observed in various squamous cell carcinomas of mucosal origin. The human telomerase RNA component (hTERC) gene encodes an RNA subunit of telomerase that maintains the length of telomeres during cellular divisions and is activated in malignant diseases. TERC gene amplifications are described in the development of various SCCs, such as those in the larynx [7], cervix, esophagus and lungs. Our results, as well those of other authors, obtained from cervical smears have demonstrated that TERC gene amplifications correlate with high-grade squamous intraepithelial lesions that lead to invasive cervical cancer. SRY-related HMG-box 2 (SOX2) gene amplifications are also described in esophageal and lung squamous cell carcinomas [8]. This intronless gene encodes a member of the SOX family of transcription factors which control the expression of a number of genes involved in embryonic development, and cooperate in the induction of pluripotent stem cells [9-11]. Recently, independent studies have identified SOX2 as a lineage-survival oncogene [12] in SCCs of the lung based on an analysis of a large set of these tumors using high-resolution and high-throughput technologies [8,13]. In a study by Bass et al. [14], amplifications of SOX2 were detected in 23% of lung SCCs and 15% of esophageal SCCs. These amplifications resulted in presumably oncogenic SOX2 overexpression. Hussenet et al. [15] and Yuan et al. [16] detected *SOX2* amplifications in lung SCCs at similar frequencies (20%). Because 3q26 amplifications are also regularly observed in oral cancer, the amplifications of the SOX2 gene could be an important diagnostic and prognostic marker. Using combined array-CGH and tissue microarray analysis Freire’s et al. [3] detected a SOX2 gene copy number gain in 52% of OSCC tumors in, which a high expression of SOX2 was suspected to be correlated with gene copy number gain.

As described in our previous study on cervical preneoplastic lesions, TERC gene amplification is a high-risk prognostic factor for cervical cancer [17] and is likely an early event in tumorigenesis in squamous cell carcinoma. Here, we investigated whether TERC and SOX2 are appropriate markers for the early detection of OSCC. The present study was designed to detect hTERC and SOX2 amplifications in OSSC exfoliative tumor cells and evaluate whether these two gene amplifications might serve as a supportive bio-marker for the non-invasive detection and diagnosis of oral and oropharyngeal SCC.

## Results

The study included 71 patients, mostly males (n = 54, 76%). Both genders had a comparable median age at diagnosis (Table 1). After study enrollment, 10 (0.14) patients died from their cancer and 14 (0.20) were lost to follow-up. All patients had histologically verified squamous cell carcinoma of the head and neck region, with tumours located mainly in the oral cavity (36 cases, 0.51; see Table 1 for details on tumour locations) or the oropharynx (27 cases, 0.38). The tumours were predominantly locally advanced cancers (48 stage IV cases, 0.68). The substantial lymph node involvement (44 cases, 0.62) may help explain the certain inconsistency between the TNM classification and disease stage data in Table 1. Distant metastases were present in the only two cases (one in the lung, one in the brain, Table 1). All patients received radical cancer treatment (surgery, chemotherapy or radiotherapy), including two patients with a histologically confirmed carcinoma in situ who had their lesions surgically removed.

**Table 1:** Clinical data of patients with OSCC

	**# (%)**	**Median age at diagnosis (range)**		**Observation time (range)**		
Women	17 (0.24)	57 (48–76) years		16 (3–27) months		
Men	54 (0.76)	58 (36–84) years		14 (3–24) months		
Deceased	10 (0.14)					
Current status unknown	14 (0.2)					
**Tumour location**	**# (%)**					
Hypopharinx	5 (0.07)					
Larynx	2 (0.03)					
Lips	1 (0.01)					
Mouth:	36 (0.51)					
Tongue	14 (0.20)					
Floor of the mouth	13 (0.18)					
Retromolar trigone	3 (0.04)					
Lower alveolus	3 (0.04)					
Buccal mucosa	2 (0.03)					
Upper alveolus	1 (0.01)					
Oropharynx	27 (0.38)					
**TNM classification**						
**Tumour**	**# (%)**	**Lymph node status**	**# (%)**	**Distant metastasis**	**# (%)**	
Tis	1 (0.01)	N0	27 (0.38)	M0	69 (0.97)	
T1	5 (0.07)	N1 or higher	44 (0.62)	M1	2 (0.03)	
T2	20 (0.28)					
T3	11 (0.25)					
T4	34 (0.48)					
**Disease stage**	**# (%)**		**Histologic grading**		**# (%)**	
0	1 (0.01)		Carcinoma in situ		2 (0.03)	
I	4 (0.06)		Grade 1		12 (0.17)	
II	11 (0.15)		Grade 2		45 (0.63)	
III	7 (0.10)		Grade 3		12 (0.17)	
IVA	35 (0.49)					
IVB	11 (0.15)					
IVC	2 (0.03)					

**Figure 1:**
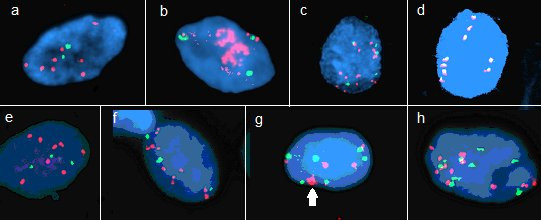
**Different level SOX2 (a, b, c, d) and TERC gene (e, f, g, h) amplifications was shown in representative cells.** The FISH pattern CEP3 : SOX2 was 2 : >4 **(a)**. Masive amplifications of SOX2 gene **(b)**. Polyploidies and amplifications of SOX2 gene **(c)**. Polyploidies of S0X2 gene in pattern 9:9. Polyploidies and amplifications of TERC gene **(e, f)**. Polyploidies of TERC gene **(g, h)**. Patches of amplifications of TERC gene in polyploid cell pointed by arrow **(g)**.

A total of 71 brush biopsies of the oropharynx and other locations in the oral cavity were analyzed by FISH using TERC, SOX2 and CEP-3 DNA probes. We considered polyploid and/or amplified cases to be abnormal. The intra and inter-tumor heterogeneity for the amplification of TERC/SOX2 locus was observed. We detected different FISH patterns in individual cases. A total of 49 of samples (69%) were considered abnormal, including 28, 10 and 5 cases in which 4–6, 6–9 and > 9 chromosomes were observed respectively. Twenty-seven of these were only polyploid (Figure 1d,g,h); 22 were polyploid and amplified, with CEP3: TERC/SOX2 FISH ratios that varied from 3:6–8, to 3–4:9–12 to 4:6–11 in the same tumor. (Figure 1b,c,e,f). Amplification only was detected in three tumors. The FISH pattern CEP3:TERC/SOX2 was 2:>4 in these tumors (Figure 1a). The over- representation of polyploidy and/or TERC/SOX2 amplification in tumor samples was statistically significant compared to controls (p = 0.01, Table 2). When cancer samples were stratified according to the disease stage the presence of polypolidy and/or amplification did not differ significantly, regardless of how the stages were combined. However, when tumors originating in the oropharynx were compared to tumours from all other locations, polyploidy and/or amplifications were observed at a significantly higher rate (p = 0.0442, Table 2).

**Table 2:** FISH analysis of TERC/SOX2 loci vs. centromere 3 in OSCC patients according to the clinical characterization

**Clinical characterization**	**SOX2/TERC amplifications/polyploidy**				**Odds ratio (95% confidence interval), p (two-tailed Fisher exact test)**		
	**Normal**	**Polyploidy only**	**Amplification and poliploidy**	**Total abnormal FISH results**	**Normal vs. total abnormal FISH results**	**Normal vs. Polyploidy only**	**Normal vs. amplification with poliploidy**
Controls	21	0	1	1	46.77 (5.91-370) 0.00	N/D^2^ (N/D^2^) 0.00	21.00 (2.56-455) 0.00
OSCC	22	27	22	49			
Stage: I, II, III	8	7	8	15	1.29 (0.39 – 4.23), 0.78	1.25 (0.41-6.03) 0.76	1.00 (0.25-4.07) 1.00
Stage: IV	14	20	14	34			
Stage: I, II	4	5	7	12	0.69 (0.17-2.77), 0.76	0.98 (0.18-5,13) 1.00	0.48 (0.09-2.34) 0.49
Stage: III, IV	18	22	15	37			
Oropharynx	6	11	10	21	2.00 (0.59-6.93), 0.29	1.83 (0.47-7.46) 0.38	2.22 (0.53-9.52) 0.34
Outside oropharynx^1^	16	16	12	28			

^1^Listed in Table 1.

^2^Not determined.

Total of 22 control samples were tested, all except one were normal. In the abnormal case the smear was taken from a healthy part of the oral cavity of a patient with diagnosed OSCC.

## Conclusion

In this study, we used two genes, SOX2 and hTERC, which are considered potential OSCC marker genes, to evaluate the FISH/cytobrush technique a screening method for cancer cell detection in smears from the oral and oropharyngeal cavity. Recurrent gene copy number aberrations are a frequent finding in OSCC and are belived to be critically involved in tumor formation. Studies using comparative genomic hybridisation and more recently, array-CGH have identified the human chromosomal region 3q21-q29 has being associated with decreased overall and disease-free survival in head and neck squamous cell carcinoma patients [[18], Choi S, Myers JN: **Molecular pathogenesis of oral squamous cell carcinoma: implications for therapy.***J Dent Res* 2008, **87**(1)**:**14–32. Review]. Amplification of the genes located in this region, particularly SOX2 and TERC, could be useful markers for malignant cell detection and could be used as prognostic markers in oral brush smears performed in the oral and oropharyngeal cavity.

We used cytobrush techniques to collect exfoliated cells from OSCC lesions. This non-invasive technique could be used as a suitable screening tool to detect suspicious oral cancer cases. Compared to tissue biopsy, this techniques is also much more agreeable to the patient. The principle of the cytobrush technique was applied as early as 1951 [19] and has been used for RNA sampling [20], although it has not been used in combination with FISH. We used these two same techniques in a study of TERC gene amplifications in cervical cancer, in which we suggested that FISH analysis for TERC amplification should be included in the routine examination of cervical cancer as a screening method [17]. The only problem in evaluating of the results obtained by this method is that with brush sampling, malignant cells are most likely not always collected in the smear. This could be the reason for at least some of the normal results obtained for our 22 cases.

Two genes were chosen for the screening, SOX2 and hTERC. An interphase FISH with a chromosome enumeration double-color DNA probe was used. Consistent with tissue biopsies and histopathological diagnoses, hTERC and SOX2 amplifications were not detected in normal oral and oropharyngeal tissues, except in one case, where the control smear was taken from the side opposite the tumor in the oral cavity, which could have been an actual positive case.

High SOX2 expression is detected in various oral squamous cell carcinomas (e.g., oral cavity and tongue); such expression is thought to be activated through gene copy number gain and is associated with poor prognosis [3,21]. Maier et al. [22] studied, various types of SCCs and detected SOX2 gene amplification in all SCC types. They found that both the primary and the metastatic tumor cell populations showed SOX2 amplification. Their data suggest that SOX2 is targeted by relatively early events during tumor development, and thus, they postulated that activity of the SOX2 protein is more important in tumor initiation than tumor progression. In our study, statistically similar levels of SOX2 and TERC amplifications occurred in all stages of the disease regardless of lymph node status and tumor classification (Tables 1 and 2).

The TERC gene is a part of the telomerase complex, which encodes the telomerase RNA template. TERC is the most frequently observed amplified oncogene in cervical precancerous lesions and linked to high-grade cervical dysplasia and cancer [17,23-25]. High telomerase expression can be explained by the amplification of the TERC gene. The results of Lui Yu et al. [7] indicate that telomerase reactivation is an early event in laryngeal carcinogenesis and is already detectable at the stage of precancerous laryngeal epithelial changes. According to our results, showing that approximately 70% of OSSCs were detected by FISH using brush smears, we would predict that this technique could also be used to analyze the precancerous lesions as a screening tool in patients with suspected oral or oropharyngeal SCC.

In all histopathologically confirmed cases the same amplification detection result was obtained for both probes that were tested; i.e., when the TERC gene was amplified, the SOX2 gene was also amplified. These results confirm the observation of recurrent amplicons of the 3q26 region, where both genes are located, as described in various types of squamous cell carcinomas [Knuutila S, Björkqvist AM, Autio K, Tarkkanen M, Wolf M, Monni O, Szymanska J, Larramendy ML, Tapper J, Pere H, El-Rifai W, Hemmer S, Wasenius VM, Vidgren V, Zhu Y: **DNA copy number amplifications in human neoplasms: review of comparative genomic hybridization studies.***Am J Pathol* 1998, **152**(5)**:**1107–1123. Review], [26]. Our results are in agreement with published array-CGH data [5] showing that extra copies of chromosome 3 regin are often detected, including all or only part of the 3q arm. Amplifications with a TERC/SOX2:CEP3 signal ratio >2 could be considered as abnormal results where true amplicons are observed, and the FISH signal occasionally appears as large dispersed areas of tested probe (Figure 1b,g). We observed abnormal polyploid cells in which almost the entire q arm of chromosome 3, including the centromere included, existed in 3, 4, 5, 6, 9 or more copies. From a prognostic point of view there is no difference between cells that are polyploid only, polyploid and amplified, or only amplified. Highly statistically significant results were detected only between all tumor cases and all controls.

Significantly different results were also obtained based on the location of the tumor. A greater number of positive cases were detected in the oropharynx compared to other locations of OSSC. This could be related to the high rate of HPV infection of esophageal carcinomas [27]. HPV infection may be responsible for the underlying chromosomal instability that can create a cellular environment where tetraploid cells can develop and proliferate. Tetraploidy often precedes the development of aneuploidy [28]. Our data show that there was a heterogeneous FISH pattern within and between tumors. These findings suggest that the higher rate of abnormal results in oropharyngeal tumors is related to polyploidies and HPV infections.

As we described in our previous study on cervical preneoplastic lesions, TERC gene amplification is a high risk prognostic factor for cervical cancer [17]. Maier et al. [22] demonstrated that SOX2 gene amplification is a common event in SCC of different organ sites, such as lung and cervix, and that it appears to be an early event in tumorigenesis. Cha et al. [29] analyzed the abundance of copy number alterations and various gene amplifications in the dysplastic transitional area of oral squamous cell carcinoma using array-CGH on fresh tissues. Those results appear to support our hypothesis that SOX2 and TERC gene amplifications are prognostic markers for OSSC and that amplifications are an early event in tumorigenesis. In the future research, these observations should be investigated on visible potentially precancerous lesions, such as erythroplakias.

In conclusion, SOX2 and TERC gene amplifications are common in all squamous cell carcinomas, and their detection in early stages could be crucial for the detection and moreaccurate prognosis of OSCCs. Our study strongly suggests that detection by FISH on cytobrushed samples could be a non-invasive screening method even before a biopsy is performed.

## Methods

Tumors were classified according to location, lymph node status and histological differentiation. Tumor cells were collected from various locations within the oral cavity: lips, tongue, mouth, larynx, hypopharynx and oropharynx, (Table 1). The controls were normal healthy individuals or buccal smears obtained from macroscopically uninvolved mucosal areas of the patients.

Brush biopsies were collected from ulcerated exophytic lesions of the oral cavity of 71 patients and 22 healthy controls. Two cytological smears were obtained from the area in question, and two were obtained from apparently normal mucosa near the lesion area using a Cytobrush Plus GT. A cytobrush cell collector was than rolled onto a glass slide. Slides were first treated with a hypotonic solution (0.0375 mol/M KCl) for 10 min at 37°C and than fixed with acetic acid and methanol (1:3) for 10 min at room temperature (RT). Slides were then dried for 30 min at 50°C and 30 min at 90°C, and than stored at −20°C until use.

Two probes were used for FISH: a combined TERC-specific DNA probe (RH17919 – 370Kb- RH10606) in red fluorescence emission and a centromere 3-specific control probe (D3Z1) in green fluorescence emission (ON hTERC 3q26/3q11 – Kreatech), or a SOX2 specific DNA probe (D3S3416 – 800Kb - D3S3957) in green combined with a centromere 3-specific probe in red (ZytoLight SPEC SOX2/CEN3 Dual Colour Probe - ZytoVision).

FISH was performed on slides pre-treated using 0.05% pepsin digestion for 30 min, fixation with 1% formaldehyde solution, and subsequent dehydration in an ethanol series (70%, 90%, 100%) and followed by air-drying. Slides were denaturated in 70% formamide for 3 min at 73°C, and probes were denaturated for 10 min at 73°C. Ten microliters of probe was applied to each slide under the 22×22 mm plastic cover slip and hybridized overnight in a moist chamber at 37°C.

The next day, the cover slips were gently removed and the slides were washed in 0.4XSSC + 0.05% Tween for 3 min at 73°C and in 2XSSC + 0.05% Tween for 2–60 s at RT. The slides were counter stained with DAPI and embedded in an anti-fadent solution. Images were acquired using an AxioImager.Z1 microscope (Zeiss, Germany) equipped with optical filters for DAPI, TRITC, and FITC (Chroma Technologies, Brattleboro, VT, USA).

The signals were evaluated by two independent screeners using an x63 objective. The signals were evaluated by screening the entire slide. Only morphologically abnormal nuclei with FISH signals for both colors were counted. Amplified and polyploid patterns were considered abnormal FISH patterns. Amplified FISH patterns had cells with two signals for CEP-3 and more than four signals for 3q26. Cells with more than 2 signals for CEP-3 and more than two signals for 3q26 with a ratio of 1:1 were considered polyploid. We evaluated at least 100 nuclei per slide and calculated the proportion of aberrant cells for each specimen. Images were analyzed using Cytovision 2.81 Version A.I. software (Applied Imaging, UK) (Figure 1).

For statistical analysis odds ratios (OR) with 95% confidence intervals (95%CI) were calculated, and the Fisher’s exact test was used to compare groups. Statistical significance was set at a P value less than 0.05.

This study was approved by the Ethics committee of the University Clinical Centre Maribor, where the study was performed, and signed informed consent was obtained from each patient.

## Competing interests

We disclose no financial competing interests:

• In the past five years we have not received any reimbursements, fees, funding, or salary from an organization that may in any way gain or lose financially from the publication of this manuscript, either now or in the future.

• We do not hold any stocks or shares in an organization that may in any way gain or lose financially from the publication of this manuscript, either now or in the future.

• We do not hold or apply for any patents relating to the content of the manuscript. We do not receive reimbursements, fees, funding, or salary from an organization that holds or has applied for patents relating to the content of the manuscript.

• We do not have any other financial competing interests.

We disclose no Non-financial competing interests:

We do not have any non-financial competing interests (political, personal, religious, ideological, academic, intellectual, commercial or any other) to declare in relation to this manuscript.

I and my co-authors ensure no competing interest.

## Authors’ contributions

NKV have made conception and design of the study, carried out the molecular cytogenetic studies, acquisition of data, analysis, interpretation of data and wrote the manuscript. BČ participated in the design of the study, collected the samples and the patients and helped to draft the manuscript. AZ carried out the molecular cytogenetic studies. BZ performed the statistical analysis. BL participated in the collecting the samples and the patients. All authors read and approved the final manuscript.
